# Effects of Physical Exercise on the Body Composition and Functionality in Individuals with Chronic Obstructive Pulmonary Disease: A Systematic Review

**DOI:** 10.3390/diagnostics14242847

**Published:** 2024-12-18

**Authors:** Daysa de Souza-Santos, Redha Taiar, José Alexandre Bachur, Luiza Torres-Nunes, Bruno Bessa Monteiro-Oliveira, Eliane de Oliveira Guedes-Aguiar, Ana Carolina Coelho-Oliveira, Vanessa Amaral Mendonça, Ana Cristina Rodrigues Lacerda, Anelise Sonza, Adérito Seixas, Mario Bernardo-Filho, Danúbia da Cunha de Sá-Caputo

**Affiliations:** 1 Laboratório de Vibrações Mecânicas e Práticas Integrativas, Departamento de Biofísica e Biometria, Instituto de Biologia Roberto Alcantara Gomes and Policlínica Universitária Piquet Carneiro, Universidade do Estado do Rio de Janeiro, Rio de Janeiro 20950-003, RJ, Brazil; daysadesouza@gmail.com (D.d.S.-S.); ltnmamae@gmail.com (L.T.-N.); bessa.oliveira@gmail.com (B.B.M.-O.); elianeguedes@gmail.com (E.d.O.G.-A.); anacarol_coelho@hotmail.com (A.C.C.-O.); bernardofilhom@gmail.com (M.B.-F.); drdanubia@gmail.com (D.d.C.d.S.-C.); 2 Mestrado Profissional em Saúde, Medicina Laboratorial e Tecnologia Forense, Universidade do Estado do Rio de Janeiro, Rio de Janeiro 20950-003, RJ, Brazil; 3 Université de Reims Champagne Ardenne, MATériaux et Ingénierie Mécanique (MATIM), CEDEX 2, 51687 Reims, France; 4 Faculdade de Medicina, Universidade de Franca, Franca 14401-426, SP, Brazil; jabachur@hotmail.com; 5 Programa de Pós-Graduação em Fisiopatologia Clínica e Experimental, Faculdade de Ciências Médicas, Universidade do Estado do Rio de Janeiro, Rio de Janeiro 20950-003, RJ, Brazil; 6 Programa de Pós-Graduação em Ciências Médicas, Faculdade de Ciências Médicas, Universidade do Estado do Rio de Janeiro, Rio de Janeiro 20950-003, RJ, Brazil; 7 Faculdade de Ciências Biológicas e da Saúde, Universidade Federal dos Vales do Jequitinhonha e Mucuri, Diamantina 39100-000, MG, Brazil; vaafisio@gmail.com (V.A.M.); lacerdaacr@gmail.com (A.C.R.L.); 8 Programa de Pós-Graduação em Ciências do Movimento Humano e Programa de Pós-Graduação em Fisioterapia, Centro de Ciências da Saúde e do Esporte, Universidade do Estado de Santa Catarina, Florianópolis 88035-001, SC, Brazil; anelise.sonza@udesc.br; 9 FP-I3ID, FP-BHS, Escola Superior de Saúde Fernando Pessoa, 4200-253 Porto, Portugal; aderito@ufp.edu.pt

**Keywords:** chronic obstructive pulmonary disease, body composition, physical exercise, functional capacity

## Abstract

Chronic obstructive pulmonary disease (COPD) is a heterogeneous condition with airflow limitation and obstructive characteristics of respiratory function. In addition, musculoskeletal dysfunction and negative changes in body composition, among other comorbidities associated with this disease, result in a low quality of life. Pulmonary rehabilitation (PR), which includes physical exercise, can positively contribute to improving the clinical conditions in individuals with COPD. **Objective**: This systematic review aims to summarize the scientific evidence on the impact of physical exercise on body composition and functionality in individuals with COPD. **Methods**: Through Boolean searches, which were carried out in the PubMed, Embase, Scopus, and Web of Science databases, 989 studies were identified. Among these studies, six were selected based on the eligibility criteria. **Results**: These studies presented a level of evidence II according to National Health and Medical Research Council criteria, with a predominance of regular methodological quality of regular according to the PEDro scale. Four studies presented a high risk of bias, and two presented a low risk of bias according to the criteria of the RoB instrument. The isolated assessment of each domain (2.0 Cochrane) presented a prevalence of 57% with a low risk of bias, followed by 23% with high risk and 20% with an uncertain risk of bias. According to the data regarding outcomes of different studies, an improvement in functional capacity through physical exercise by individuals with COPD was observed. Simultaneously there were reports regarding body composition demonstrating no significant improvement in fat-free mass and fat mass. **Conclusions**: Improvements in the body composition and functionality in individuals with COPD can promote a better quality of life, favoring the management of this population. This systematic review presents evidence of the potential benefit of improving the functionality of individuals with COPD. Other aspects of the health of this population were also improved, such as quality of life. However, the results related to body composition are inconclusive regarding a decrease in fat mass and an increase in fat-free mass. Therefore, studies of higher quality should be developed to evaluate the effects of physical exercise on the body composition of individuals with COPD.

## 1. Introduction

Smoking is a chronic condition included in the International Statistical Classification of Diseases and Related Health Problems (ICD-10), which, according to the World Health Organization (WHO), is caused by nicotine dependence. Prolonged use of tobacco products can contribute to irreversible damage to the lungs, leading to the clinical condition known as chronic obstructive pulmonary disease (COPD) [1,2,3,4]. Smoking and inhaling toxic particles and gases from air pollution are the main causes of COPD [1].

COPD is a heterogeneous, common, preventable, and treatable condition characterized by persistent respiratory symptoms and airflow limitation due to airway and/or alveolar anomalies [1]. In addition to chronic inflammation of the airways, active inflammatory cells and increased plasma levels of pro-inflammatory cytokines in systemic circulation have been observed, which, together with oxidative stress, contribute to nutritional changes and musculoskeletal dysfunctions observed in individuals with COPD [1,5,6]. These comorbidities associated with this disease result in a low quality of life for individuals with COPD [5].

Lean mass and visceral fat area (VFA) are associated with inflammatory parameters stimulated in individuals with COPD [7]. Loss of body mass and muscle mass are common problems for individuals with severe or very severe COPD [1]. Furthermore, individuals with obesity have the worst prognosis for COPD [8].

Musculoskeletal dysfunction can occur even in mild COPD and contributes significantly to physical inactivity, leading to airflow obstruction. Physical inactivity impairs functional status and, consequently, may be associated with a decrease in the quality of life in individuals with COPD, in addition to contributing to the loss of muscle mass and changes in body composition [9].

Studies demonstrate that both muscle mass and skeletal muscle strength in individuals with COPD are significantly reduced when compared to healthy individuals of the same age [10]. Furthermore, there is evidence that musculoskeletal myopathy contributes to the severe functional disability experienced by these patients [10]. Therefore, individuals with COPD may experience a reduction in the capacity and volume of activities of daily living, which is one of the main factors that contribute to their morbidity [11]. In addition to this comorbidity resulting from reduced functional capacity, musculoskeletal disorders, other comorbidities, such as chronic diseases, heart failure, and malnutrition, may be present in COPD and strongly contribute to a greater possibility of hospitalization and disease progression [12].

Severe shortness of breath leads to limited daily physical activity in individuals with COPD, who restrict themselves from performing physical activities to reduce dyspnea episodes [13]. According to the Global Initiative for Obstructive Lung Disease (GOLD) guidelines, pulmonary rehabilitation (PR) would be indicated for individuals with COPD [1]. PR is part of non-pharmacological interventions. Non-pharmacological interventions include smoking cessation, education about the disease, changes in lifestyle habits, and also physical exercise [1]. Physical exercise is one of the interventions that is relevant to improving the health status of these individuals [1].

It is assumed that physical exercise is an important therapeutic intervention in the treatment of individuals with COPD [10], with the potential to significantly improve physical function and health-related quality of life (HRQoL) [1], as well as respiratory function. Continuous or interval resistance exercises can positively influence the quality of life of individuals with COPD, as can strength training associated with aerobic training improve functional capacity in these patients [1], in addition to increases in lean mass and reductions in fat mass in these individuals [14].

Different types of physical exercises are described in the literature for the treatment of individuals with COPD, within the context of PR programs. These exercises can be performed with different intensities, time, and loads [15]. Authors who conducted their studies in aquatic and terrestrial environments noticed increased functionality, including in the distance covered and the time pedaled on the cycle ergometer [16]. In their review, Spruit et al. (2016) describe the historical trajectory of the implementation of physical training (aerobic and anaerobic exercises) in the treatment of individuals with COPD, stating that this population obtained a response of greater functional performance [17], thus pointing out physical training as the cornerstone in pulmonary rehabilitation in these individuals [17]. In terms of body composition, some studies that implemented exercise protocols with aerobic training resulted in a decrease in fat mass and an increase in fat-free mass [14]. Other authors reported an increase in fat-free mass after a two-month intervention [18].

However, these positive effects depend on the type of physical exercise proposed, as well as its respective frequency, intensity, and duration [11]. For some years, it has been stated that there is no evidence regarding the ideal exercise format [17,19]. Therefore, the objective of this systematic review is to summarize the scientific evidence on the impact of physical exercise on body composition and functionality in individuals with COPD.

## 2. Materials and Methods

This review was conducted based on the Preferred Reporting Items for Systematic Reviews and Meta-Analyses (PRISMA) recommendations [20], and the protocol was registered in the International Prospective Register of Systematic Reviews (PROSPERO) under CRD42022333706.

Given the absence of a systematic review, with or without meta-analysis, related to the aforementioned objective, a systematic review of randomized clinical studies relevant to the effects of physical exercise on the body composition and functionality of individuals with COPD was developed.

Research question: This systematic review aims to answer the following question: What is the scientific evidence on the effects of physical exercise on the body composition and functionality of individuals with COPD?

The PICOS strategy was used to define the components of the research question: Participants (P) = individuals with COPD; Interventions (I) = physical exercise; Comparators (C) = control group without physical exercise or different types of physical exercise alone; Outcome (O) = effects on body composition and functionality; Study design (S) = randomized controlled trial (RCT).

Eligibility criteria: The inclusion criteria were as follows: (i) Investigations into the effects of physical exercise on the body composition and functionality of individuals with COPD; (ii) RCT study; (iii) publications regardless of the year; (iv) physical exercise alone or combined with other therapies, and (v) publications in English. Exclusion criteria were as follows: (i) themes different from the topic covered, research letters, conference abstracts, languages other than English, editorials, and communications; (ii) studies focusing on polyunsaturated fatty acids, heart rate variability, anabolic steroids, or gut microbiota; (iii) studies involving inflammatory markers, cachexia, sarcopenia, elderly people, osteoarthritis, endocrine diseases, or diabetes mellitus; (iv) studies related to other diseases or individuals with obesity; (v) studies with exercise associated with other therapies, such as nutritional support or medicinal herbs, and (vi) studies with other interventions that did not involve physical exercise.

Search strategies: An electronic search was performed in February 2023 and redone in July 2024 in the PubMed, Embase, Scopus, and Web of Science databases, and it was conducted by two independent reviewers (DSS and ACCO).

Study Selection: All the studies from Table 1 were exported to the software-as-a-service Rayyan (SaaS or a web application) [21] and Word^®^ (Microsoft, Redmond, WA, USA) documents as Rayyan and Word documents. Duplicates were manually removed using Rayyan by two authors (DSS and LTN), and a third reviewer (BBMO) was consulted. The selection of studies was blinded among the evaluators. The review was finalized following three steps. Records were identified through database searches and reference screening (Identification), titles and abstracts were independently selected, and irrelevant studies were excluded based on eligibility criteria (Screening). Relevant full texts were screened for eligibility, and all relevant studies were included in the systematic review. The same researchers were responsible for extracting data from the included studies. Data related to study information: author and year (aim, gender of participants, groups, age and mean, body mass index, functional capacity assessment, pulmonary measures, and conclusion), and intervention protocol (study, participants, type of exercise, exercise protocol, time and periodicity and intensity of exercise).

Assessments of methodological quality (MQ), level of evidence (LE), and risk of bias (RB) of selected articles: The studies were evaluated independently by two reviewers (DSS and LTN), and in case of disagreement, a third reviewer (BBMO) was consulted to reach a consensus. It is emphasized that all members of the evaluating team were previously trained in the use of different tools.

The methodological quality assessment was carried out based on the Physiotherapy Evidence Database (PEDro) scale, which consists of a checklist with eleven items established based on “expert consensus”. This tool is used in physiotherapy and rehabilitation centers and consists of 11 items. A score from 0 to 10 is used to define quality. Publications were classified into ‘high’ methodological quality (scores equal to or greater than seven), ‘fair’ methodological quality (scores from five to six), and ‘poor’ methodological quality (scores equal to or less than four) [22,23,24,25].

To assess the level of evidence (LE), the criteria proposed by the National Health and Medical Research Council Hierarchy of Evidence (NHMRC) were used. This hierarchy of evidence (NHMRC) was developed as part of a series of comprehensive manuals outlining methods for evaluating evidence and for developing and disseminating clinical practice guidelines. These manuals recommended that the body of evidence should be evaluated along three dimensions: (1) strength, (2) effect size, and (3) clinical relevance [26]. They are highlighted in Table 2.

The risk of bias (RB) in the RCT was determined based on the use of the Cochrane Collaboration tool, in which each domain can be classified as low risk, uncertain risk, or high risk of bias. These are represented, respectively, by the colors green, yellow, and red. To assess the risk of bias in RCTs, the RoB 2.0 tool (Cochrane’s revised risk of bias tool for randomized trials) is currently the tool recommended by the Cochrane Collaboration. According to the tool, for each study result of interest, five domains were evaluated relating to possible biases in the study. The five domains are (1) bias in the randomization process, (2) deviations from the intended intervention, (3) bias due to missing data, (4) bias in outcome measurement, and (5) bias in outcome reporting. Each domain can be classified as low risk of bias, few concerns, or a high risk of bias [27].

The extraction of data on the effects observed in each study is shown in Table 3, consisting of the study, objective, gender of participants, exercise intervention groups, age, body mass index, body mass and its averages, assessment instruments for functionality, lung assessment instruments, and completion of each study. The extraction of data on the intervention protocols of each included study is shown in Table 4, consisting of studies, study participants, intervention protocols, exercise protocols, exercise frequency, and intensity.

## 3. Results

**Figure 1:**
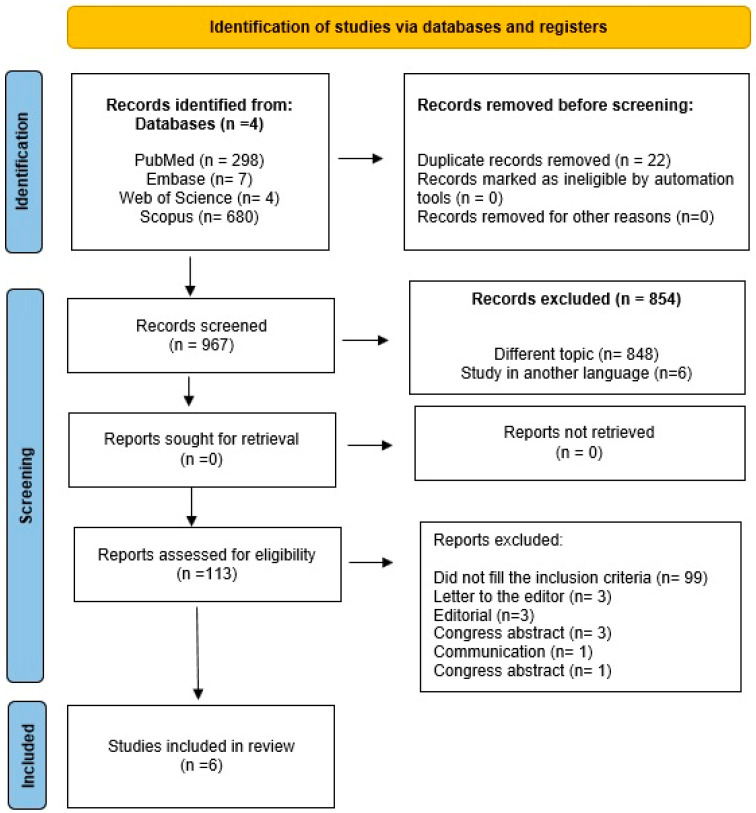
PRISMA flowchart for study selection.

Firstly, 989 studies were found (PubMed = 298, Web of Science = 4, Embase = 7, and Scopus = 680). Then, 22 duplicates were removed, and 967 studies were screened. Nine hundred and sixty-one (*n* = 961) studies were excluded because they covered different topics, were in languages other than English, were conference abstracts, research letters, communications, editorials, or did not meet the inclusion criteria. Eligibility criteria were applied, and six articles met the inclusion criteria and were included in this systematic review. The full process of determining eligibility for the studies, from collecting articles to selecting articles for data extraction, is highlighted in the flowchart, as proposed in PRISMA (Figure 1).

### 3.1. Body Composition Assessment

Considering the findings presented in Table 3*,* measuring instruments used for evaluating body composition were electrical impedance [30,33], fat-free mass (FFM), FFM index (FFMI = FFM/height^2^) [33], dual-energy X-ray absorptiometry (DEXA) [28,29,32], and a body scanner [31]. From the examination, the regional body composition of the lower limbs (fat content and fat-free mass) was expressed as a percentage of body mass used in the analyses [28].

### 3.2. Functionality Assessment

To assess functionality, the following evaluations were used: (i) the 6-min walk test (6 MWT) [5,28,30,31,33]; (ii) the incremental walking test (ISWT) [30]; (iii) the resistance test, the timed up-and-go (TUG) test [28], the ergometric cycle, and the constant work rate cycling endurance test (CWRT) [32]; (iv) the treadmill resistance test with constant load (TEnd) [33]; (v)The sit and reach test [31]; (vi) London Chest Activity of Daily Living (LCADL) [30], the maximum number of sitting and standing during one minute, and the number of steps [29]; (vii) sit on the floor and, with your knees straight, place your hand over your hand and stretch as far as possible without bending your knees, and the best of three attempts was recorded [31].

### 3.3. Interventions

Considering the findings presented in Table 4, of the selected studies, one applied 12 weeks of physical exercise consisting of strength exercises (bodybuilding), low-intensity exercises, and combined exercises (strength + low intensity), with 35 participants [33]; one evaluated 120 participants who were subjected to 8 weeks of intervention with muscular strength exercises and low-frequency (15 Hz) and high-frequency (75 Hz) electrical stimulation in the quadriceps and gastrocnemius muscles in both legs [32]. One study subjected 28 male participants to 28 weeks of intervention and 14 weeks of follow-up. The exercise protocols in one group included aerobic exercises with flexibility exercises, balance exercises, Nordic walking, or weightless circuit training, and the combined therapy group included aerobic exercises with resistance exercises [31]. One study evaluated 36 participants in 6 months using the same physical training protocol as the exercise program, including walking and exercises for the upper limbs, such as warm-up exercises, strength training for the lower and upper limbs, and stretching (upper limbs, lower limbs, cervical, and trunk) performed in the water and on land [30]. Another study evaluated 78 participants over 13 weeks, using high-load leg press exercises (10 repetition maximum, RM), low-load contralateral leg (30 RM) exercises, and combined knee extension exercises [29].

One study applied 12 weeks of training in 20 participants [28]. The training involved an eccentric and concentric cycle ergometer protocol to gradually increase the volume and intensity of physical training, as highlighted in Table 4.

### 3.4. Main Findings

The reported findings obtained an improvement in muscle strength and functional capacity (6 MWT), peak isokinetic torque of the quadriceps, and muscle strength associated with resistance [32]. However, body mass index and fat mass index did not change significantly [32]. The study reported an improvement in functional exercise capacity, peripheral muscle strength, and maximum exercise capacity, and there was no significant change in body composition [30]. The study by Inostroza et al. (2022) reported progressive improvement in fat-free mass in the lower limbs, improvement in the 6 MWT distance, and reduction in time for the TUG, as well as the stair climbing and descending test [28]. Rinaldo et al. (2017) found improvements in 6 MWT, muscle strength, and flexibility, and considered the types of interventions good for preventing an increase in fat mass and a decrease in lean mass [31]. Molmen et al. (2021) reported that they found marked signs of improvement in muscle strength, muscle mass and quality, single-leg endurance, performance in the 6-min step test, performance of sitting and standing in 1 min, maximum workload achieved during bipedal cycling, and a non-significant reduction in lean mass for the COPD group [29]. In the Dourado et al. (2009) study on exercise tolerance, the muscular strength training group and the combined training group demonstrated a significant improvement in basal peripheral muscular strength after training in all values of 1-RM (repetition maximum), as well as an increase in the 6-min walking distance and endurance time [33]. Regarding body composition, there was no significant change [33].

In the studies included, there are similarities among the intervention protocols according to the types of exercises performed. Refs. [29,30,31,32,33] evaluated the effect of anaerobic exercises. Dourado et al. (2009) and Felcar et al. (2017) were the only authors who evaluated their protocol with exercises for both upper and lower limbs [30,33]. Anaerobic exercise, aerobic exercise, and stretching was also examined [30,31]. Inostroza et al. (2022) evaluated only aerobic exercises [28].

Studies that evaluated anaerobic exercise described improvements in physical exercise performance, strength, muscular endurance, and balance [29,30,31,32,33]. Studies that analyzed combined exercises, such as anaerobic, aerobic, and stretching exercises, generated good results [30,31].

Table 3 shows the data extracted from each selected study, considering author and year, objectives, demographics (sample size, age, gender, body mass, and body mass index), functional capacity assessment, pulmonary measures, and clinical conclusions.

### 3.5. Study Population

Three hundred and seventeen individuals were evaluated in this systematic review, of which two hundred and fifty-nine were diagnosed with COPD and fifty-eight were healthy. Regarding gender, the COPD group had up to one hundred and thirty-five males and ninety-eight females, and the healthy group had up to twenty-one males and thirty-seven females [29]. One study used only males [31].

The mean age of individuals with COPD ranged from 61 to 71 years. Healthy individuals were 67 ± 4 years old. The lowest average age was reported by Dourado et al. (2009), which was 61.3 ± 8.8 years in the strength training group (ST group) [33], and the highest average age was 71.1 ± 10.3 years in the control group (CONC group) [28].

### 3.6. Methodological Quality

**Figure 2:**
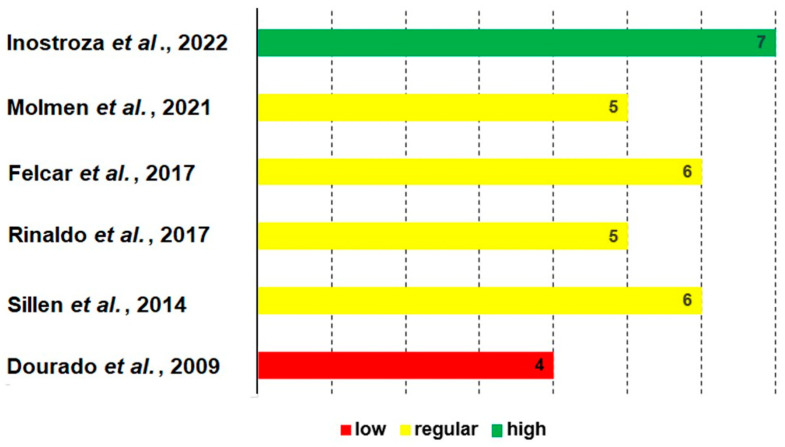
Pedro scale scores [28,29,30,31,32,33]. Green = high methodological quality; yellow = regular methodological quality, and red = low methodological quality.

According to the data from the assessment of the level of methodological quality based on the PEDro scale (Figure 2), it was observed that one study was considered low [33], four were fair [29,30,34,35], and one was considered high [28]. Thus, 66% presented a moderate level of methodological quality, 17% with a high level, and 17% with a low level.

### 3.7. Risk of Bias

**Figure 3:**
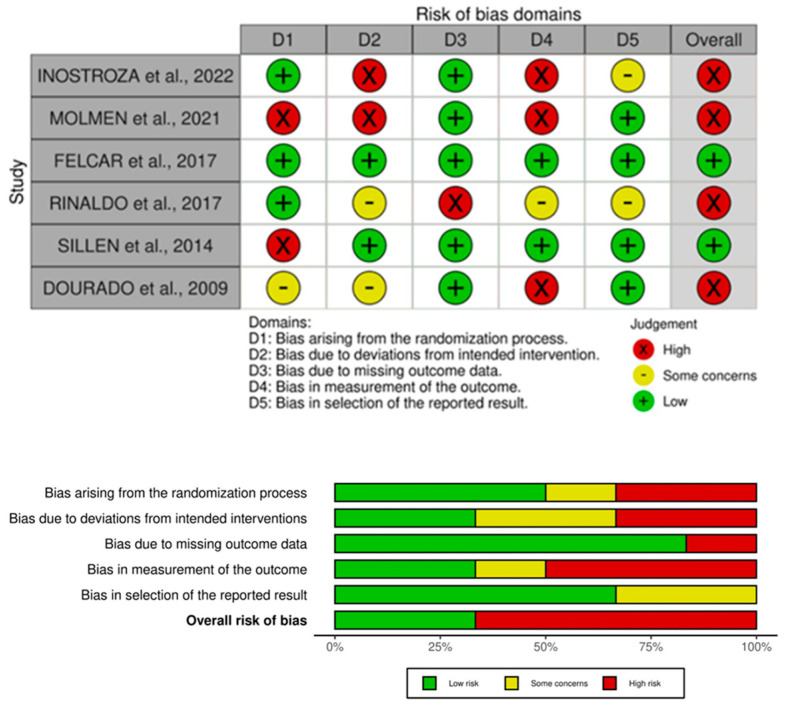
Risk of bias in randomized trials included based on Cochrane Reviews [28,29,30,31,32,33]. Domains (D): D1 = bias arising from the randomization process; D2 = bias due to deviations from the intended interventions; D3 = bias due to missing outcome data; D4 = bias in the measurement of the outcome; D5 = bias in the selection of the reported result. Green = low risk of bias; yellow = unclear risk of bias, and red = high risk of bias [27].

The analysis of the risk of bias using the Cochrane risk of bias tool (Figure 3) showed that four studies were classified as high risk of bias [28,29,31,33] and two studies were classified as low risk of bias [30,32].

**Figure 4:**
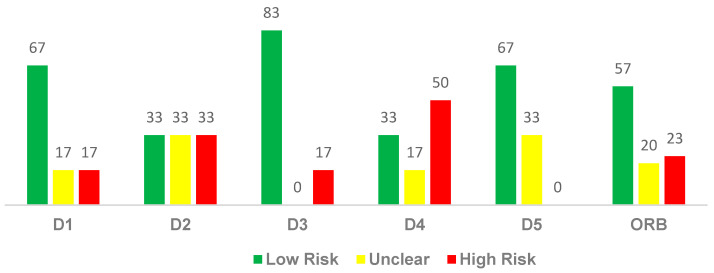
Evaluation of the risk of bias levels by domain in the sample universe. Domains (D): D1 = bias arising from the randomization process; D2 = bias due to deviations from the intended intervention; D3 = bias due to missing outcome data; D4 = bias in the measurement of the outcome; D5 = bias in the selection of the reported result, and ORB = overall risk of bias.

When analyzing the different domains of risk of bias (Figure 4), a predominance of low risk of bias is observed at around 57%, while ‘uncertain’ and ‘high risk’ are 20% and 23%, respectively.

## 4. Discussion

The objective of this systematic review was to summarize the scientific evidence on the impact of physical exercise on body composition and functionality in individuals with COPD. This current systematic review suggests that physical exercise is a non-pharmacological intervention that can improve body composition, decrease fat mass, increase fat-free mass, and enhance functional capacity in individuals with COPD, improving strength, muscular resistance, flexibility, balance, and distance and walking speed

### 4.1. Body Composition (Fat Mass and Fat-Free Mass)

Nine articles were found that agreed with three of the authors included in this study. There was no significant change in body composition, with a reduction in fat mass and an increase in fat-free mass. However, these results may have occurred due to differences in study protocols, types of exercises, time and frequency, and intensity of exercises.

One study did not find any significant change in either the free mass index or the fat-free mass at the end of the program [30]. According to this study, it was verified that there was no association between changes in body composition (fat mass, fat mass index, fat-free mass, and fat-free mass index) and some moderate physical exercise after 6 days a week using a physical exercise tracker [13].

One study analyzed breathing techniques, arm training, and nutritional supplements, and after 1 year of intervention, they demonstrated no change in the fat-free mass index [36]. However, this study described an increase in lean mass in the lower limbs, suggesting efficacy only in the eccentric cycling group [28]. Reinforcing what was previously stated in this study, there was a significant decrease in the percentage of body fat and a tendency toward an increase in lean body mass content [14]. In a meta-analysis, it was found that studies that analyzed eccentric and concentric cycling were equally effective in reducing body fat percentage [18]. The protocol duration of 1 to 2 months was shown to be similar to the protocol longer than 2 months [18].

In the Nápolis study, where the intervention of high-intensity electrical stimulation was applied for 6 weeks, there was an improvement in fat-free mass [37]. This agrees with our included study, which had high-intensity electrostimulation in its protocol [32].

This study did not show a significant improvement in fat mass and demonstrated significant improvement in lean mass after the intervention [29]. In the exercise protocol, they observed that participants did not obtain improvements at baseline or after the intervention, neither in lean mass nor in fat mass. In the follow-up assessment, a slight worsening in body composition was seen [31].

In Franssen’s study, where they analyzed a combination of resistance, strength, and cycle ergometry exercises in a normal-weight population with COPD, they reported that their participants achieved changes in body composition after eight weeks of physical training, resulting in a significant increase in body weight, leading to an increase in their fat-free mass and a slight decrease in fat mass [1].

Another study did not find any significant change in lean mass index [33]. Similarly, another study applied a resistance training protocol with muscular strength training, and after the exercise intervention, it was found that there was no significant difference between fat mass and lean mass, neither in the arm nor in the leg [10]. In agreement, they found no significant improvement in lean mass or fat mass [38]. These findings corroborate the conclusion that the result of muscular strength is observed in individuals who performed muscular strength training, and the increase in total lean mass would only be observed when these individuals were in a rehabilitation program with lung disease associated with conditioning physiotherapy, nutritional support, or supplementation with specific agents [5]. These results are in line with studies that combined nutritional supplements and physical training, which found an increase in body composition and fat mass [39].

In a cross-sectional study, individuals diagnosed with COPD with and without sarcopenia were compared, and it was possible to identify that sarcopenic individuals with COPD have lower fat mass and lean mass [40].

Body composition is considered important for the prognosis of COPD [12]. Depletion of fat mass and fat-free mass was present in most individuals diagnosed with the disease [12]. In a review, it was stated that interventions in body composition are extremely relevant for an individualized approach for the population with chronic respiratory impairment, to contribute to physical condition [41]. Therefore, it was reported that nutritional support was part of pulmonary rehabilitation [41].

### 4.2. Functional Capacity

Nine studies are in agreement with two of the included studies.

There was an improvement in the eccentric cycling (ECC) group, suggesting that the improvement in functional performance and rapid force production was effective in this group after the intervention [28]. This study compared the effect of a 3-month resistance training intervention program, and after five years, the participants undertook a 3-month strength training program. These participants improved all forms of physical function in the resistance training program, and the strength training program improvement was demonstrated only in 6 WMD [10].

In a study where they analyzed the effects of full-body exercise training (a combination of resistance and strength exercises and cycle ergometry) in the normal-weight population with COPD, they highlighted that there was a significant improvement in exercise performance during the training period [1]. They also stated that age is a predictor of baseline functional capacity in these individuals diagnosed with COPD, independent of lung function and fat-free mass [1].

The narrative review corroborates the use of whole-body vibration exercise (EVCI) as another differential for pulmonary rehabilitation, resulting in improved functional performance of the lower limbs and quality of life [42]. According to the authors, who also analyzed the EVCI intervention, they found similar improvements concerning the increase in exercise capacity, aligning with the findings of this study [43].

The investigation by Braumer et al. (2023) points out the association of multi-nutrient supplements with high-intensity physical training and demonstrates improvements in lower limb muscle strength and exercise capacity when compared to placebo [44].

In a systematic review, it was shown that PR with at least 4 weeks of exercise intervention results in statistically and clinically significant gains in quality of life and functional capacity. In addition, it reduces hospital readmissions [45].

It is possible to suggest that PR is safe for individuals with COPD and associated comorbidities, as they obtain responses of significant and clinically relevant improvements in their health status and functional capacity [17]. After 12 weeks of high-intensity training, a study with individuals with COPD showed improvements in functional capacity [11].

In the randomized study, participants performed two intervention programs: one of moderate to intense activity and the other of low intensity [46]. They showed that a moderate-intensity training program is effective in improving exercise capacity and shoulder abduction strength at 4 months, and they found a difference between the groups of 26.6 m in the 6 MWT and considered the significance not only statistically but also clinically [46].

### 4.3. Functionality (Tests and Scales)

Five of the included studies evaluated the 6-min walk test and found an increase in the 6 WMD distance [28,30,31,32,33]. As with the seven studies, the 6-min walk test is widely used in studies due to its easy application [47].

One included study used the 1-min sit-to-stand (STS), 6-min step, cycling performance, sitting, and maximum load achieved with bipedal cycling tests, all of which showed improvements [29]. Improvements were also observed in balance [34], endurance [33], and the functional test (TUG) [28]. The total Canadian Occupational Measure (COPM) score for problematic activities of daily living (ADLs) improved in all groups compared to baseline [32].

In the systematic review, it was demonstrated that the variables 6 MWT and ESWT increased after the intervention [48], which is in agreement with our study [30]. This study reported improvement in maximal exercise capacity in the incremental walk test and also improvement in the endurance walk test [49].

One study also reported that after 24 sessions, there was a significant increase in the aquatic group in the 6 MWT [50]. However, there was no significant difference between the groups (land, water, and control) for the 6 WMT [50]. An investigation of a pulmonary telerehabilitation program (three times a week for 10 weeks, weekly exercise volume of 105 min) and a conventional pulmonary program (two times a week for 10 weeks)—including warm-up, resistance training, and cool-down, with a total protocol time of 22 weeks [51]—found a significant and statistical increase in the 6-min walk time only in the telerehabilitation group after 22 weeks of intervention [51].

Consistent with the findings of this systematic review with meta-analysis, researchers have stated that both interval exercise and continuous exercise provide better quality of exercise capacity assessed through the 6 WMT, recovery of symptoms, and quality of life in individuals with COPD [11].

In one study, aerobic, strength, and flexibility exercises were applied in 20 sessions, and there was an improvement in the ergometric test with constant load; there was an improvement in the LCADL scale, which is different from our studies [30]; where was also improvement in the incremental effort test and in the 6 MWT [35].

According to a cross-sectional study, individuals with COPD with and without sarcopenia were compared through functional tests, where it was possible to identify that sarcopenic individuals with COPD presented lower functional performance for the 6 MWT and 5-sit-to-stand tests (STS) [40].

This research confirmed the increase in the distance in the 6 MWT, indicating that aerobic physical activity (exercise, cycling, or walking) of moderate intensity is more efficient for quadriceps muscle dysfunction in COPD [38]. Furthermore, they suggested that PR should include aerobic training [38].

In this study, they evaluated two physical training programs (PWT), one with low-intensity and the other with moderate-intensity exercise, in patients with mild-to-moderate COPD [46]. They found an improvement in exercise capacity and shoulder abduction strength in 4 months, and a difference between the groups of 26.6 m in the 6 MWT [46]. Thus, the results are considered not only statistically but also clinically significant [46].

In the study by Larsson et al. (2018), where the SPPB test was studied, which is composed of three subtests, their findings showed no change in the balance subtest, but there were significant improvements in the -MGS subtests (timed four-meter walk test at usual walking speed) and in the 5-STS tests (timed test of five repetitions from sitting to standing on a chair—performed as fast as possible) after the completion of the RP program (strength and resistance training) [52]. They also pointed out that higher SPPB scores were correlated with the improvement of the 6 WMD distance [52].

A systematic review and meta-analysis showed that interval and continuous exercises lead to better exercise capacity measured for 6 MWD [11]. Corroborating the findings of this study, an increase in isometric muscle strength of the quadriceps was demonstrated in another study [14].

### 4.4. Functionality (Neurostimulation Electrical)

This was the only study included that investigated, in its intervention protocol, exercises with high-intensity electrical neurostimulation (HF-NMES) 75 Hz, low-intensity (LF-NMES) 15 Hz, and strength training [32]. It demonstrated a significant increase in the peak isokinetic torque of the quadriceps after HF-NMES and strength training [32]. There was an increase in muscular resistance in the three groups [32]. According to the *American Journal of Respiratory and Critical Care Medicine*, electrical neurostimulation is a promising modality for improving lower limb function, being useful in individuals with a diagnosis of more severe COPD [21].

In this study, HF-NMES demonstrated improved load and work cycle, increasing from 16 to 33% over the 6 weeks of the protocol, and exercise capacity also improved significantly in individuals who had preserved fat-free mass and greater tolerance to electrical stimulation [37]. Resistance, strength, and high-frequency neurostimulation training for 24 weeks (72 sessions) had significant increases in terms of exercise tolerance, lower limb strength, and static and dynamic balance, in addition to functional capacity. In the group that received exercise training combined with electrical neurostimulation, both groups showed improvements in functional exercise capacity and peripheral muscle strength after 3 months of training [53]. As in this study, they reported improvement in 6 MWT distance that was noted after 6 weeks of high-intensity (50 Hz) neuromuscular electrical stimulation in both quadriceps [54]. In line with our findings, it also states that high-frequency electrical neurostimulation is as effective as strength training [54].

Supporting the included studies, in the findings of this narrative review, high-frequency intervention achieved an increase in quadriceps muscle function, 6 MWT distance, endurance time, quality of life, activity of daily living, dyspnea, exercise capacity, and health status [44].

### 4.5. Functionality (Muscle Strength)

All included articles confirmed that their protocols obtained muscle strength findings. Only in one study was there no result by group, but by participants. There were two participants in the eccentric cycling group and three in the concentric cycling group, which resulted in a 20% improvement in muscle strength [28]. In a randomized study, it was stated that PR is an exercise-based therapy known to increase quadriceps strength, which results in a decrease in hospital emergency room visits and mortality [34].

This study demonstrated an increase in isometric quadriceps muscle strength [14]. Unlike our findings, exercise capacity improved, but there were no significant changes in peripheral muscle strength [48].

According to a review, where exercises in the aquatic environment were compared with the terrestrial environment, it was seen that there was an improvement in maximum knee flexion strength in both groups; however, knee extension strength was only improved in the land-based training group [55]. In a semi-randomized study, aquatic intervention and land-based training for 12 weeks improved muscular endurance in aquatic training [16].

An eight-week protocol of exercise training combined with resistance, strength, and cycle ergometry exercises in the COPD population claimed that participants demonstrated a 30% improvement in quadriceps strength [1]. They also suggested that exercise may act as an anabolic stimulus for this population [1].

A significant increase in percentage changes in grip strength was found in the randomized clinical trial among the three groups analyzed (aerobic exercise group combined with recreational activity, aerobic exercise group combined with muscular strength training, and the control group, which received no exercise) [56].

A narrative review validates whole-body vibration exercise (WBVE) as a form of physical exercise for individuals with COPD, improving their functional performance and quality of life [42].

A review that describes musculoskeletal dysfunction in the COPD population confirms the importance of pulmonary rehabilitation in muscle recovery [8].

### 4.6. Limitations

Although important findings are presented, there are limitations in this systematic review, such as the use of four databases. In addition, there are limitations regarding the included studies, the study design, the heterogeneity of the physical exercise protocols, the quality of the studies, and the different ways of assessing body composition and functionality. Therefore, it is necessary to carry out studies with better methodological quality so that more robust evidence can be promoted on the subject. We also consider as limiting factors of the present study the fact that it was not possible to constitute a sufficient sample set to fully meet the necessary criteria for evaluating publication bias through sensitivity analysis, as well as the pattern of symmetry between the data from the different studies evaluated.

### 4.7. Strength

The strength of this systematic review is to demonstrate the effects and benefits of physical exercise on body composition and functionality in individuals with COPD and reinforce the importance of this non-pharmacological intervention within an RP program. Improvements in the body composition and functionality of individuals with COPD are important in the management of this condition.

Considering the facts and perspectives of this study, physical exercise is an important non-pharmacological intervention to be considered in the management of individuals with COPD, leading to better management of this condition.

## 5. Conclusions

This systematic review presents evidence of the potential benefit of improving functionality (strength, muscular endurance, and exercise capacity) in individuals with COPD. Other aspects of the health of this population were also improved, such as quality of life.

However, the results related to body composition are inconclusive regarding the decrease in fat mass and the increase in fat-free mass.

Therefore, it is suggested that higher-quality studies be developed to evaluate the effects of physical exercise on the body composition of individuals with COPD.

## Figures and Tables

**Table 1:** Electronic databases and their respective strings.

Database	Boolean Strategy
PubMed	((COPD OR chronic obstructive pulmonary disease) AND (body composition) AND (exercise OR physical activity) AND (functionality)).
Embase	(‘copd’/exp OR copd OR ‘chronic obstructive pulmonary disease’ OR (chronic AND obstructive AND pulmonary AND (‘disease’/exp OR disease))) AND (‘body composition’/exp OR ‘body composition’ OR ((‘body’/exp OR body) AND composition)) AND (‘exercise’/exp OR exercise OR ‘physical activity’/exp OR ‘physical activity’ OR (physical AND (‘activity’/exp OR activity))) AND functionality
Scopus	(((copd OR “chronic obstructive pulmonary disease”) AND (“body composition”) AND (exercise OR “physical activity”) AND (functionality)))
Web of Science	((copd OR “chronic obstructive pulmonary disease”) AND (“body composition”) AND (exercise OR physical activity) AND (functionality))

**Table 2:** Level of evidence by the National Health and Medical Research Council Hierarchy of Evidence (NHMRC).

Level of Evidence	Concept
I	✓ The systematic review of level II studies.
II	✓ Randomized controlled trial.
III-1	✓ The pseudo-randomized controlled trial (alternate allocation, crossover study, or some other similar method).
III-2	✓ The comparative study with concurrent controls (non-randomized experimental trial, cohort study, case-control study, or interrupted time series with a control group).
III-3	✓ The comparative study without concurrent control (historical control, two or more single-arm studies, or interrupted time series without a parallel control group).
IV	✓ The case series with either post-test or pretest/post-test outcomes.

**Table 3:** Aims and outcomes of the selected studies.

Study	Aim	Gender of Participants, Groups, Age and Mean	Functional Capacity Assessment	Pulmonary Measures	Conclusion
Inostroza et al., 2022 [28]	To compare the effects of ECC and CONC training on muscle function, body composition, functional performance, and QOL in moderate COPD individuals.	Male/female (10/10) ECC Age 68.2 ± 10.0 (52–86) years BMI (kg/m^2^) 29.1 ± 5.4 (24.0–32.8) CONC Age 71.1 ± 10.3 (51–83) years BMI (kg/m^2^) 28.1 ± 5.9 (19.6–40.2)	- Cycle ergometer - 6 MWT - MVC strength and its rate of force development - TUG - Stairs ascending and descending walking time	- SGRQ	Three times greater work was produced in eccentric than concentric cycling training at the same rating of perceived exertion, which resulted in fewer increases in heart rate and greater oxygen saturation. Remarkably, eccentric cycling training improved lower limb fat-free mass, RFD, 6 MWT, and TUG to a greater extent than concentric cycling training.
Molmen et al., 2021 [29]	To investigate the assumed negative effects of COPD on health- and muscle-related responsiveness to resistance training using a healthy control-based translational approach.	Male/female (33/45) COPD Age 69 ± 5 years Body mass (kg) 73 (18) BMI (kg/m^2^) 25 (5) Healthy Age 67 ± 4 years Body mass (kg) 76 (16) BMI (kg/m^2^) 26 (5)	- 6 min step test (maximal number of steps) - 1 min sit-to-stand test (maximal number) repetitions at 50% of 1-RM knee extension. - One-legged endurance	- SF-36 - CAT	The 13-week resistance training program was well-tolerated by subjects with COPD and led to pronounced improvements in a range of health, muscle function, and biological variables, resembling or exceeding those seen in healthy individuals, with some outcome measures even showing indices of more beneficial adaptations in COPD individuals with a more severe diagnosis. COPD was thus not associated with impaired responsiveness to resistance exercise training, which rather served a potent measure to relieve disease-related pathophysiologies. However, they did not obtain significant results in body composition regarding the reduction of fat mass and the increase in fat-free mass.
Felcar et al., 2017 [30]	To compare the effects of 2 similar 6-month protocols of high-intensity exercise training, in water and on land, in COPD individuals.	Male/female (23/13) LG Age 68 ± 8 years BMI (kg/m^2^) 26 ± 5 WG Age 69 ± 9 years BMI (kg/m^2^) 26 ± 5	- 6 MWT - 1-RM - ISWT - LCADL	- MRC - HANDS - CRDQ	High-intensity exercise training in water generates similar effects compared with training on land in patients with moderate-to-severe COPD, making it an equally beneficial therapeutic option for this population. Regarding the findings on body composition, they did not find significant results in the reduction of fat mass and the increase in fat-free mass.
Rinaldo et al., 2017 [31]	To achieve these aims, the protocol of the physical activity education program included explicit instructions, work examples, and role-playing with an accompanying training manual to ensure quality and consistency in the techniques adopted and behaviors displayed.	Male CT Age 66.2 ± 4.2 years EDU Age 66.1 ± 4.5 years	- 6 MWT - Upper body and bilateral lower limb strength (1-RM) - The sit-and-reach - Timed one-leg stance test	- MRF-26	Our results demonstrated that both EDU and CT walking ability, balance, CMO, quality of life, and adherence also improved similarly to training in patients with stable COPD, although such parameters returned to baseline values after 14 weeks of follow-up. Furthermore, muscle strength improved after training in CT alone, and, similar to the aforementioned parameters, it returned to baseline after the follow-up period. Regarding body composition, they reported an increase in fat-free mass.
Sillen et al., 2014 [32]	To prospectively study the efficacy of HF-NMES (75 Hz), LF-NMES (15 Hz), or strength training in severely dyspnoeic COPD individuals with quadriceps muscle weakness at baseline.	Male/female (62/58) HF-NMES Age 64.4 ± 1.3 years BMI (kg/m^2^) 24.1 ± 0.8 LF-NMES Age 66.2 ± 1.3 years BMI (kg/m^2^) 25.5 ± 0.8 ST Age 64.0 ± 1.3 years BMI (kg/m^2^) 24.9 ± 0.8	- 6 MWT - CWRT	- HANDS - SGRQ Cardiopulmonary exercise test	HF-NMES is equally effective as strength training in severely dyspnoeic individuals with COPD and muscle weakness in strengthening the quadriceps muscles and thus may be a good alternative for this particular group of patients. HF-NMES, LF-NMES, and strength training were effective in improving exercise performance in severely dyspnoeic individuals with COPD and quadriceps weakness. However, there were significant results in lower limb fat-free mass.
Dourado et al., 2009 [33]	To compare three different physical exercise programs in COPD individuals: moderate to ST, LGT, and CT.	Male/female (26/9) ST Age 61.3 ± 8.8 years Body mass (kg) 72 ± 14.5 BMI (kg/m^2^) 26.8 ± 4.4 LGT Age 62.1 ± 10 years Body mass (kg) 68.9 ± 13.6 BMI (kg/m^2^) 27.1 ± 5.8 CT Age 65.4 ± 9.2 years Body mass (kg) 64.8 ± 13 BMI (kg/m^2^) 23.6 ± 5.4	- 6 MWT - TEnd - Handgrip strength - Peripheral muscle strength	- SGRQ - Airways Questionnaire 20 - BDI	The benefits of physical conditioning for healthy status in patients with COPD seem to be independent of the modality or intensity of the exercise training undertaken. Despite this, there were no significant results in body composition in reducing fat mass and increasing fat-free mass.

RCT = randomized clinical trial N = Number; with strength training; QOL = quality of life; 6 MWT = 6-min walking test; TEnd = constant workload treadmill endurance test; CET = cycle endurance time; SGRQ = St George’s Respiratory Questionnaire; FCT: functional circuit training; 1-RM = one-repetition maximum; BDI = baseline dyspnea index; TUG = timed up-and-go test; ST = strength training; LGT = low-intensity general training; CT = combined training groups; CONC = Concentric cycling; ECC = Eccentric cycling; CWRT = Constant work-rate cycling endurance test; HF-NMES = high-frequency neuromuscular electrical stimulation; LF-NMES = low-frequency neuromuscular electrical stimulation; COPD = chronic obstructive pulmonary disease; HADS = Hospital Anxiety and Depression Scale; LCADL = London Chest Activity Daily Living scale; CRDQ = Chronic Respiratory Disease Questionnaire; HRQL = health-related quality of life; CAT = COPD Assessment Test; SF-36 = The Short Form Health Survey; mMRC = modified medical research; Council Dyspnea Scale; PASE = Physical Activity Scale for the Elderly; SES = Chronic Disease Self-Efficacy Scale; BODE = body-mass index, airflow obstruction, dyspnea, and exercise capacity index; MRF-26 = Maugeri Respiratory Failure Questionnaire.

**Table 4:** Intervention protocol table of included studies.

Study	Participants	Type of Exercise	Exercise Protocol	Time and Periodicity	Intensity of Exercise
Inostroza et al., 2022 [28]	GI—*n* = 10 GII—*n* = 10	Aerobic Exercise	Recumbent cycle ergometer - GI = Concentric group - GII = Eccentric group	- 12 weeks: 2 training sessions; first 2 weeks and 3 sessions per week for the following weeks. - 7 weeks of training: cycling with 2-min rest (3 × 10 min) - Last 5 weeks: cycling with 2 min rest (2 × 15 min)	- 2nd week—perceived exertion rate of 11 (Borg) - 3rd week—perceived exertion rate of 12 - 4th to 12th week—perceived exertion rate of 13
Molmen et al., 2021 [29]	GI—*n* = 20 GII—*n* = 58	Anaerobic exercise	The leg exercises—leg press, knee extension, and knee flexion—were performed unilaterally in that consecutive order, with one of the legs of each participant being randomly assigned. GI = COPD group GII = HEALTHY group	- 13 weeks - For each exercise, all 3× for one leg were conducted before the other leg was exercised.	- 60 min - 3 × 10 RM - (high-load) - Contralateral - leg 3 × 30 RM (low-load)
Felcar et al., 2017 [30]	GI—*n* = 20 GII—*n* = 16	Anaerobic exercise; Aerobic exercise; Stretching	Endurance training cycling and walking; Strength training for lower limbs (quadriceps) and upper limbs (biceps and triceps); Stretching of lower and upper limbs, cervical, and trunk muscles. GI = WG group GII = LG group	6 months: - (60 sessions) - 3 months, 3× week - 3 months, 2× week - 8 educational sessions	- 1-h session Each training session involved warming up (1 min of walking and metabolic exercises for the upper limbs). - Pace dictated by sound stimulus from a metronome
Rinaldo et al., 2017 [31]	GI—*n* = 12 GII—*n* = 12	Aerobic exercise Stretching Anaerobic exercise	Aerobic exercise with flexibility and balance exercises, Nordic walking, or weight-free exercises in circuit training. Weight-free exercises: Squats, lunges, crunches, and tailored push-ups were performed as circuit training; Endurance exercises: Cycling, treadmill walking, or using an upper-limb ergometer. Each training session ended with flexibility and balance exercises using proprioceptive boards. GI = CT group GII = EDU group	28 weeks: 1 set × 8 repetitions; Nordic Walking 10 to 15× Weeks 1–5: sessions 3 days/week; Weeks 6–10: sessions 2 days/week and self-directed training sessions 1 day/week; Weeks 10–14: sessions 1 day/week and self-directed training sessions 2 days/week; Weeks 15–28: self-directed training sessions 3 days/week. CT program fitness exercises 3 days/week with at least 1 day in between each session.	Each supervised training session lasted 60 min and included 3 alternating exercises. Aerobic classes: Intensity from 3 to 4; they included weight-free exercises; 4× of lower limb; Endurance exercises: 30 min; Intensity from 3 to 4 weeks according to 1 RM test results.
Sillen et al., 2014 [32]	GI—*n* = 29 GII—*n* = 33 GIII—*n* = 29	Low and high-intensity electrostimulation; Anaerobic exercise.	After a continuous 3-min warm-up at 5 Hz. GI = LF-NMES (15 Hz) group GII = HF-NMES (75 Hz) group The quadriceps and calf muscles of both legs were stimulated electrically. GIII = Strength training group Bilateral leg extension and bilateral leg press exercises.	8 weeks, 2× a day, 5× week. 4 × 8 repetitions for each exercise with at least 2 min of recovery between each set.	The training load was set to increase by 5% every 2 weeks. Both exercises started at 70% of 1-RM; The intensity was adjusted to individual tolerance during each 18-min session.
Dourado et al., 2009 [33]	GI—*n* = 11 GII—*n* = 13 GIII—*n* = 11	Anaerobic exercise	GI = ST group Performed on weight training machines. GII = LGT group Low-intensity resistance training with free weights: diagonal upper-limb strength training, biceps curl; shoulder flexor strength training; pectoralis strength training; triceps Curl. On exercise mats: hip abductor strength training; hip and lumbar extensor strength training; abdominal exercises. On parallel bars: thoracic and upper-limb strength training; squats; diagonal upper-limb strength training. GIII = CT group Walking at a self-low-intensity resistance training with free weights, on exercise mats, and parallel bars.	60 min ST: 3 × 12 repetitions with a 2 min rest between sets LGT: 30 min of walking and an additional 30 min of low-intensity resistance training. Free weights: from 20 to 25 repetitions. Exercise mats: from 25 to 50 repetitions. Parallel bars: from 15 to 25 repetitions. CT: 30 min of ST with only 2 × 8 repetitions; The remaining 30 min were devoted to LGT at half the volume. ST: 3 × 12 repetitions with a 2-min rest between sets;	ST: with a workload at 50–80% of that achieved on the 1-RM. LGT: self-determined intensity; high number of repetitions with a low load. CT: with a workload at 50–80% of 1-RM.

CONC = concentric cycling; ECC = eccentric cycling; HF-NMES = high-frequency neuromuscular electrical stimulation; LF-NMES = low-frequency neuromuscular electrical stimulation; COPD = chronic obstructive pulmonary disease; WG = water group; LG = land group; ST = strength training; LGT = low-intensity general training; CT = combined training groups.

## Data Availability

All stages of the studies followed the Prisma protocol and all data are available.
